# Oral administration of ferulic acid or ethyl ferulate attenuates retinal damage in sodium iodate-induced retinal degeneration mice

**DOI:** 10.1038/s41598-020-65673-y

**Published:** 2020-05-26

**Authors:** Masayuki Kohno, Kunihiro Musashi, Hanako Ohashi Ikeda, Tomohisa Horibe, Aki Matsumoto, Koji Kawakami

**Affiliations:** 1 Department of Pharmacoepidemiology, Graduate School of Medicine and Public Health, Kyoto University, Kyoto, Japan; 2 Medical Platform Co. Ltd., Osaka, Japan; 3 Department of Ophthalmology and Visual Sciences, Graduate School of Medicine, Kyoto University, Kyoto, Japan

**Keywords:** Diseases, Health care, Medical research, Molecular medicine

## Abstract

Epidemiological studies indicate that the daily intake of antioxidants from a traditional Asian diet reduces the risk of developing age-related macular degeneration. Many of the phytochemicals that are abundant in whole grains exhibit a wide variety of biological activity such as antioxidant, anti-inflammatory, and neuroprotective effects. Ferulic acid (FA) is a phenolic acid found in vegetables and grains that has therapeutic potential for diabetes mellitus, Alzheimer’s disease, and other diseases. We investigated the retinal protective effect of FA in a sodium iodate (NaIO_3_)-induced model of retinal degeneration. In a human retinal pigment epithelial cell line, FA attenuated H_2_O_2_-induced injury and lipopolysaccharide- or 7-ketocholesterol-induced inflammation. In mice, the oral administration of FA or its analog, ethyl ferulate, attenuated the morphological and functional features of NaIO_3_-induced retinal degeneration according to optical coherence tomography and electroretinography. Our results demonstrate that the oral administration of FA provides protective effects to the retina, suggesting that the intake of FA as a daily supplement or daily healthy diet containing rich vegetables and whole grains may prevent age-related macular degeneration.

## Introduction

Age-related macular degeneration (AMD) is the primary cause of blindness in the elderly in Western countries^1^. AMD is a progressive neurodegenerative disease affecting the macula that results in irreversible visual impairment. The late stages of AMD can be classified into (i) wet and (ii) dry forms: (i) choroidal neovascularization with newly formed blood vessels in the macular region; and (ii) geographic atrophy with major loss of the retinal pigment epithelium and choriocapillaris^2^. Wet AMD can be treated by repeated intravitreal injections of anti-vascular endothelial growth factor agents, but no effective treatment exists for dry AMD. AMD is a multifactorial disease, with aging, genetic factors, oxidative stress, light exposure, and inflammation contributing to its pathogenesis^3^.

Several modifiable lifestyle factors are associated with a lower prevalence of AMD, including certain healthy diets, physical activity, and not smoking^4^. The Western diet has been associated with an increased risk of developing AMD^5^. Conversely, the traditional Asian diet and the Mediterranean diet are associated with decreased risks of developing AMD^5,6^. Some clinical studies have shown that the progression of AMD can be slowed with lutein/zeaxanthin supplements^7,8^.

Plants produce various phytochemicals as antioxidants in order to protect themselves against ultraviolet radiation from sunlight. Many phytochemicals, such as polyphenols and phenolic acids, are included in vegetables, fruits, grains, and tea leaves, and have been proposed for the treatment of various chronic and intractable diseases^9^.

Whole grains are rich sources of various phytochemicals, and their consumption has been associated with a reduced risk of developing major chronic diseases such as cancer, diabetes, and cardiovascular disease^10^. In fact, brown rice and one of its components, γ-oryzanol, improve glucose metabolism and attenuate the preference for dietary fat in mice fed a high-fat diet^11^. Rice bran is produced by milling rice and is an important source of various phytochemicals, and it is especially rich in the phenolic compounds γ-oryzanol and ferulic acid (FA)^12^. γ-Oryzanol is a combined ester of FA and phytosterols. FA is the main metabolite of γ-oryzanol and has been proposed as a potential treatment for many diseases including cancer, diabetes, cardiovascular disease, and Alzheimer’s disease (AD)^13^. AD is a late-onset, neurodegenerative disease that shares several clinical and pathological features with AMD. There is growing evidence of epidemiological, molecular, and clinical links between AD and AMD^14^.

FA causes the unpleasant bitter and astringent taste of alcoholic beverages such as *sake*; however, its analog, ethyl ferulate (EF), contained in *sake* and *sake* mash, has a more pleasant taste than FA^15^. EF reportedly has antioxidant and anti-inflammatory effects *in vitro* and *in vivo* by oral administration^16,17^. Because EF has an ester group, it is more lipophilic and has higher cell membrane and blood-brain barrier permeability than FA. In this study, we demonstrated the protective effect of FA and EF in a mouse model of retinal degeneration.

## Results

### FA attenuates H_2_O_2_-induced injury in ARPE-19 cells

**Figure 1:**
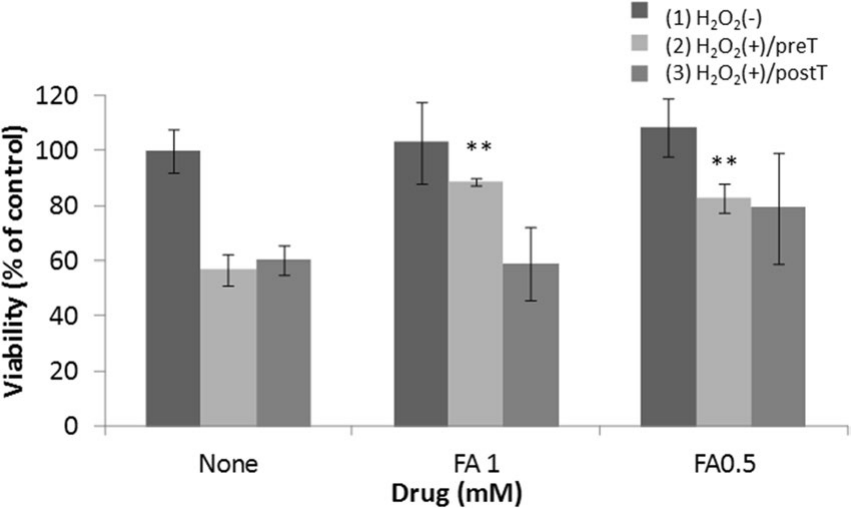
Effect of FA on H_2_O_2_-induced injury in ARPE-19 cells. (1) ARPE-19 cells were treated with FA for 3 days without exposure to H_2_O_2_. (2) ARPE-19 cells were pretreated without or with FA for 1 h before a 1-h exposure to 0.3 mM H_2_O_2_. The cells were incubated in fresh medium for 3 days. (3) ARPE-19 cells were exposed to 0.3 mM H_2_O_2_ for 1 h. The medium was replaced, FA was added, and the cells were incubated for 3 days. Data represent the mean ± standard deviation (SD) of technical replicates from triplicate determinations, and the assay was repeated three times with similar results to confirm the result. **p < 0.01 vs. None (Dunnett’s test).

From some reports, FA has been shown to be effective over a relatively wide concentration range, and based on this information, we determined the FA concentration of the *vitro* experiment^13,18,19^. To investigate whether FA could attenuate oxidative stress in the retina, we assessed whether FA reduced H_2_O_2_-induced injury in a human retinal pigment epithelial cell line (ARPE-19 cells). Furthermore, we examined differences in the cytoprotective effect of FA before and after H_2_O_2_ exposure for 1 h. FA (0.5 or 1 mM) did not affect the viability of ARPE-19 cells (column 1 in Fig. 1), and 0.4 mM H_2_O_2_ exposure for 1 h caused a 40–50% decrease in cell viability. Treatment with FA attenuated cell damage, and pretreatment with FA was more effective than post-treatment with FA (columns 2 and 3 in Fig. 1).

### FA attenuates lipopolysaccharide (LPS)- or 7-ketocholesterol (7KCh)-induced inflammation in ARPE-19 cells

**Figure 2:**
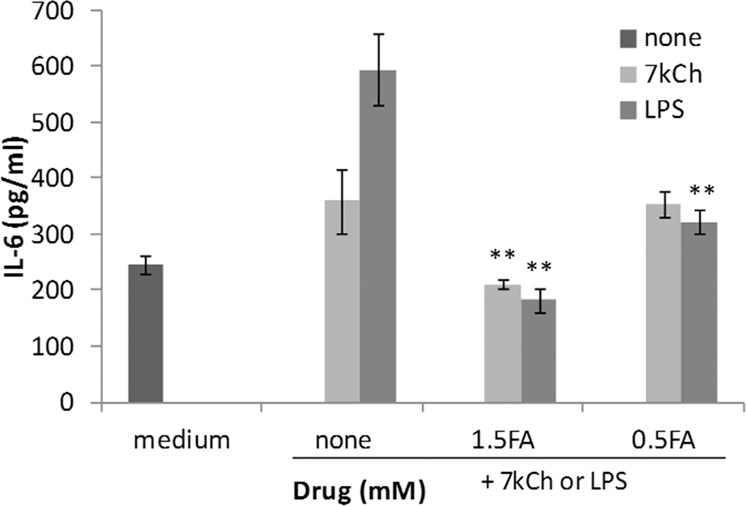
Effect of FA on LPS- or 7KCh-induced inflammation in ARPE-19 cells. ARPE-19 cells were cultured with LPS or 7KCh for 48 h with or without 0.5 or 1.5 mM FA. Data represent the mean ± SD of technical replicates from triplicate determinations, and the assay was repeated three times with similar results to confirm the result. **p < 0.01 vs. None (Dunnett’s test).

Many phytochemicals, including FA, reportedly have anti-inflammatory effects, and FA has been shown to be effective over a relatively wide concentration range in anti-inflammatory assay^20,21^. To investigate whether FA could attenuate inflammation in the retina, we assessed whether it could reduce interleukin (IL)-6 production in ARPE-19 cells exposed to LPS or 7KCh. Exposure to LPS or 7KCh for 48 h increased IL-6 production in ARPE-19 cells, and treatment with FA attenuated its production (Fig. 2).

### Oral treatment with FA attenuates N-methyl-N-nitrosourea (MNU)-induced retinal degeneration in mice

For pharmacological induction of experimental retinal degeneration, MNU or sodium iodate (NaIO_3_) has been used^22,23^. First, we investigated the effect of FA against MNU-induced retinal degeneration. Mice (ddY) were injected with MNU interperitoneally at a dose of 70 mg/kg body weight. FA (10 mg/kg/day) was administered orally using a feeding needle once a day from the first day of MNU injection to 4 days after. Quantification of the outer nuclear layer (ONL) and total retina demonstrated retinal degeneration by day 4 in mice treated with MNU compared to control, and treatment with oral FA attenuated the MNU-induced damage (Supplementary Fig. S1). Next, we confirmed reproducibility of the effect of FA against MNU-induced retinal degeneration. Mice were injected with MNU interperitoneally at a dose of 60 mg/kg body weight. FA was administered orally using a feeding needle twice a day or interperitoneally once a day from the first day of MNU injection to 4 days after. Quantification of the ONL and total retina demonstrated retinal degeneration by day 4 in mice treated with MNU compared to control, and treatment with FA attenuated the MNU-induced damage (Supplementary Fig. S2). After conducting the second animal experiment, MNU was discontinued at Sigma-Aldrich and no longer available. We obtained another MNU (containing water) from Toronto Research Chemicals, but its damage activity was weaker than the activity of the sigma-MNU (Supplementary Fig. S3). In such a state, it was judged that it would be difficult to evaluate the efficacy of FA, and the retinal degeneration-inducing agent was changed from MNU to NaIO_3_.

### Oral pretreatment with FA attenuates NaIO_3_-induced retinal degeneration in mice

**Figure 3:**
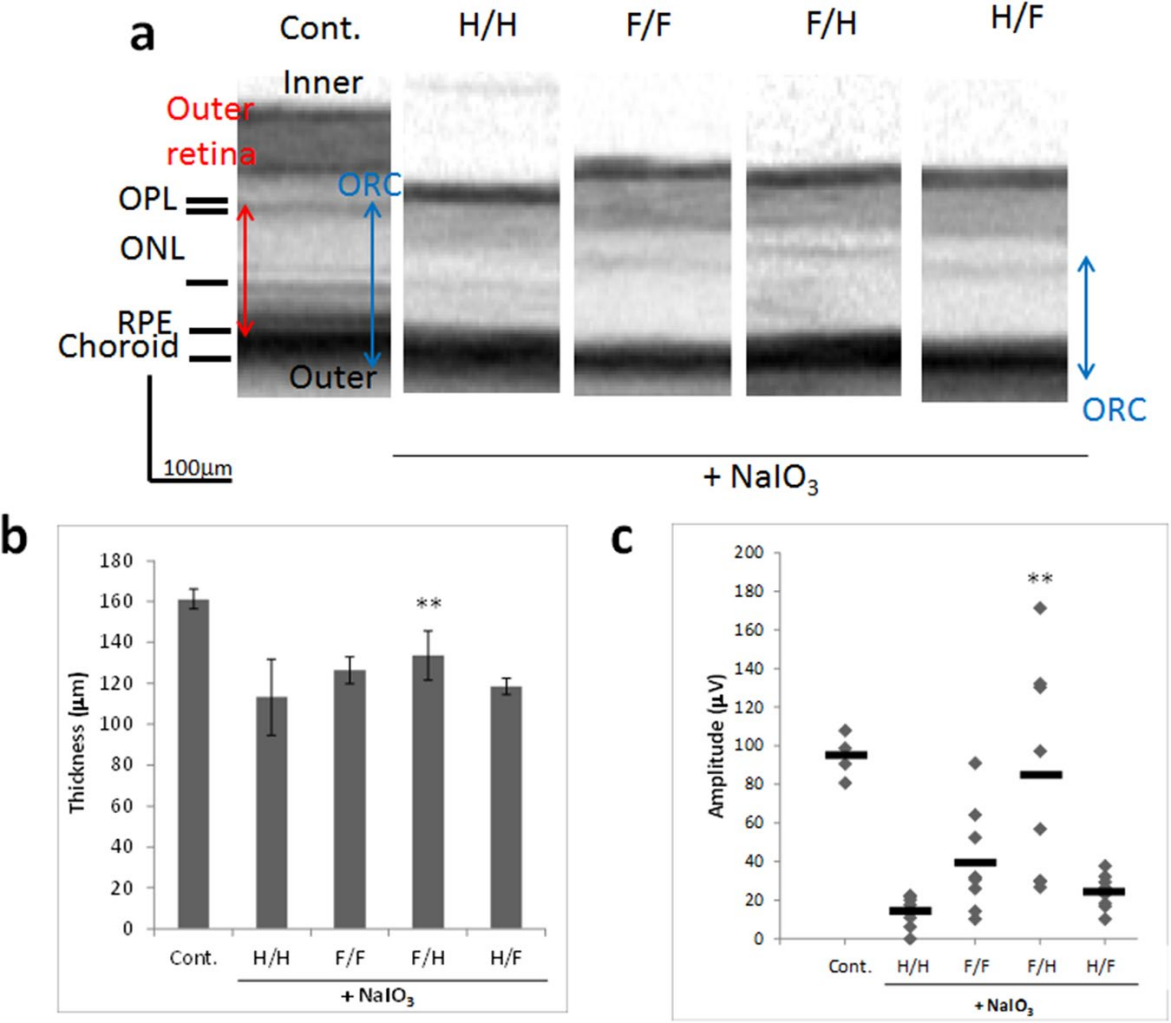
Oral treatment with FA attenuates NaIO_3_-induced retinal degeneration in mice. (**a**) Representative SD-OCT images of control mice and mice treated with various combinations of H_2_O or FA for 15 days after NaIO_3_ administration. SD-OCT images showing the cross-sectional retinal thickness of mice in each group. The thickness of the ORC was measured from the OPL until the choroid. H:H_2_O; F: FA. (**b**) Quantitative analysis of ORC thickness by SD-OCT for each group. Data are shown as the mean ± SD (4 eyes in the control group; 8 eyes in the NaIO_3_-treated groups). **p < 0.01 vs. H/H (Dunnett’s test). (**c**) Oral pretreatment with FA attenuates NaIO_3_-injured visual function at 15 days after NaIO_3_ injection. Photopic ERG responses of b-waves elicited by light at an intensity of 30 cds/m^2^ were recorded at 15 days after NaIO_3_ injection. **p < 0.01 vs. H/H (Dunnett’s test).

Next, we investigated the effect of oral FA pre- or post-treatment on NaIO_3_-induced retinal degeneration. C57BL/6 mice were injected with NaIO_3_ interperitoneally at a dose of 40 mg/kg body weight. FA was administered orally using a water bottle (30 mg/kg/day), and we divided FA treatment into pre-treatment from 3 days before NaIO_3_ injection to 1 day after and post-treatment from 1 day after NaIO_3_ injection to 15 days after (Supplementary Table S1). Spectral-domain optical coherence tomography (SD-OCT) images revealed that the cumulative thickness of the outer retina (OR, from the outer plexiform layer to the retinal pigment epithelium) and the choroid layer (ORC) was significantly reduced at 15 days after administration of NaIO_3_ (H/H group, pretreatment with H_2_O/post-treatment with H_2_O) compared with the control group. Pre-treatment with FA attenuated the NaIO_3_-induced retinal damage and suppressed the thinning of the ORC (Fig. 3a,b). On day 15, b-wave amplitude was strongly reduced in NaIO_3_-exposed retina compared with the control group. Pre-treatment with FA (F/H) attenuated NaIO_3_-induced retinal dysfunction and increased electroretinogram (ERG) responses (Fig. 3c), and more effectively attenuated the NaIO_3_-induced retinal damage compared to post-treatment with FA (H/F).

### EF attenuates H_2_O_2_-induced injury in ARPE-19 cells

**Figure 4:**
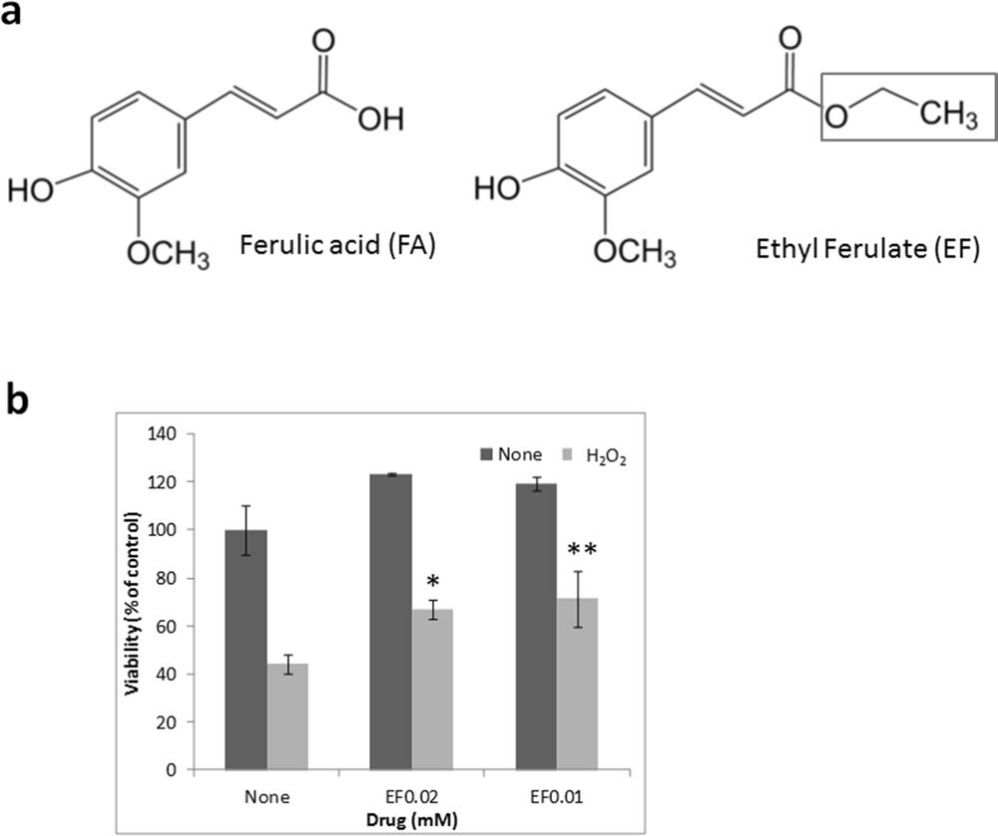
Effect of EF on H_2_O_2_-induced injury in ARPE-19 cells. (**a**) Chemical structures of FA and EF. Square in EF indicate the different portion for chemical structure from FA. (**b**) ARPE-19 cells were treated with EF for 24 h before a 1-h exposure to 0.4 mM H_2_O_2_. The cells were incubated in fresh medium for 24 h. Data represent the mean ± SD of technical replicates from triplicate determinations, and the assay was repeated three times with similar results to confirm the result. **p < 0.01, *p < 0.05 vs. None (Dunnett’s test).

To investigate whether the FA analog EF could attenuate oxidative stress in the retina, we assessed whether FA could reduce H_2_O_2_-induced injury in ARPE-19 cells (Fig. 4a). EF (0.02 or 0.01 mM) did not affect the viability of ARPE-19 cells. Exposure to 0.4 mM H_2_O_2_ for 1 h caused a 50–60% loss in cell viability, and treatment with EF attenuated this cell damage (Fig. 4b).

### Oral treatment with EF attenuates NaIO_3_-induced retinal degeneration in mice

Next, we investigated the effect of EF treatment on NaIO_3_-induced retinal degeneration. C57BL/6 mice were injected with NaIO_3_ interperitoneally at a dose of 40 mg/kg body weight. EF was administered orally using a water bottle from 3 days before NaIO_3_ injection to 14 days after (30 mg/kg/day).

**Figure 5:**
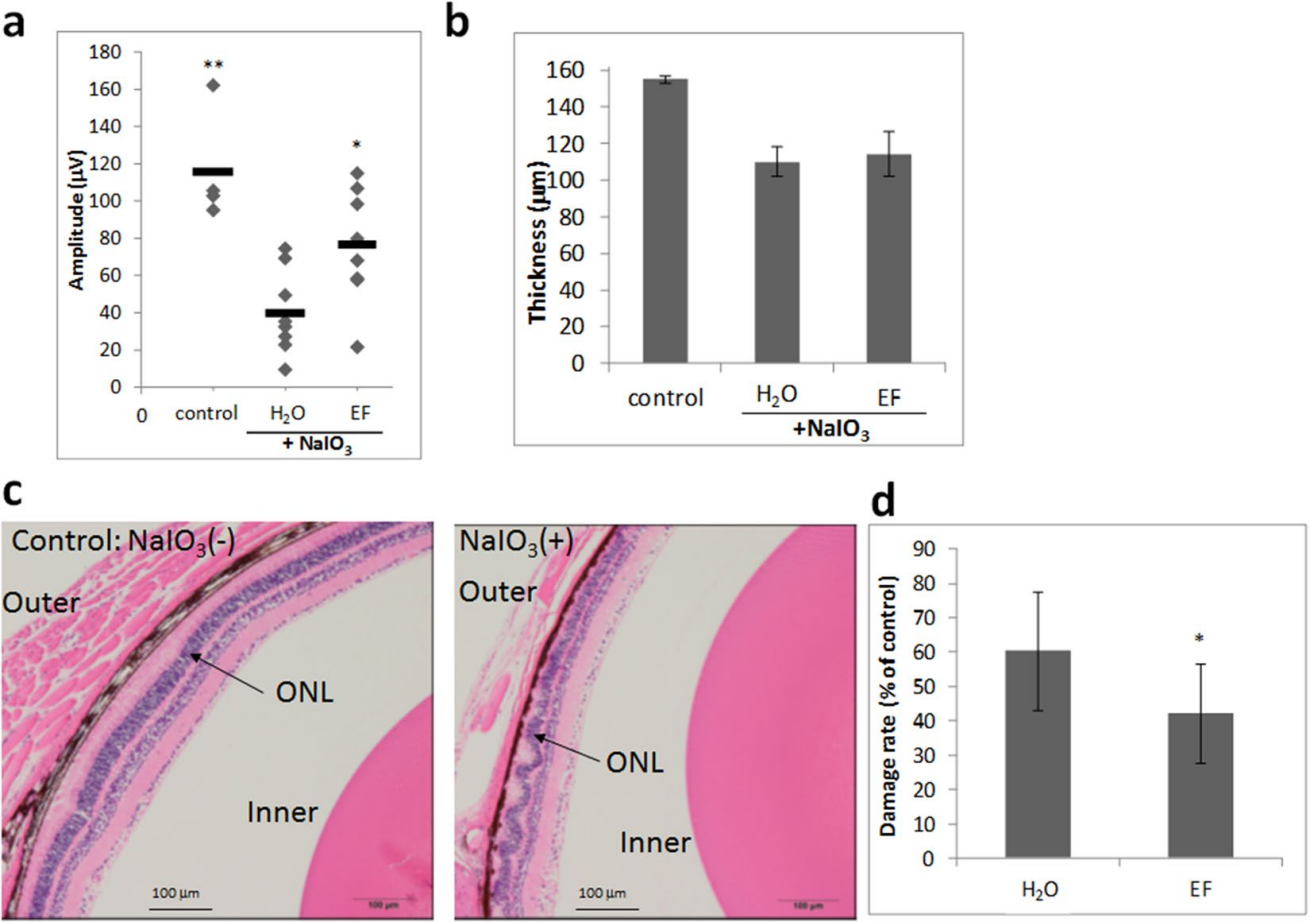
Oral treatment with EF attenuates NaIO_3_-induced retinal degeneration in mice. (**a**) Oral pretreatment with EF attenuates NaIO_3_-injured photoreceptor function. Photopic ERG responses of b-waves elicited by light at an intensity of 30 cds/m^2^ were recorded at 14 days after NaIO_3_ injection. **p < 0.01, * p < 0.05 versus H_2_O (Dunnett’s test). (**b**) Quantitative analysis of ORC thickness by SD-OCT for each group at 14 days after NaIO_3_ injection. (**c,d**) Evaluation of the damage in ONL lesions of HE-stained retinal sections of NaIO_3_-induced mice treated with H_2_O or EF. (**c**) Representative images of HE-stained retinal sections of mice treated without or with NaIO_3_. (**d**) Damage rate of ONL lesions in NaIO_3_-induced mice treated with H_2_O or EF. Data are shown as the mean ± SD (4 eyes in the control group; 8 eyes in the NaIO_3_-treated group). *p < 0.05 vs. H_2_O (Student’s *t*-test).

On day 14, b-wave amplitude was strongly reduced in NaIO_3_-exposed retina compared with the control group, and treatment with EF attenuated NaIO_3_-induced retinal dysfunction and increased ERG responses (Fig. 5a). SD-OCT images revealed that the thickness of the ORC was significantly reduced at 14 days after NaIO_3_ injection compared with the control group. However, the thickness of the ORC in SD-OCT images of NaIO_3_-injected mice did not differ between H_2_O and EF administration (Fig. 5b). To validate the retinal imaging results, degeneration profiles were measured in histological sections of the retina collected at 14 days after NaIO_3_ injection. In the retina of NaIO_3_-injected mice, obvious damage was found in the ONL, cone and rod layer, and retinal pigmented epithelium, forming a series of irregular folds in the outer retina (Fig. 5c). The rate of irregularities (cumulative retinal length exhibiting irregular ONL vs. total retinal length) in histological sections cutting across the optic disc was measured^18^. Treatment with EF attenuated the NaIO_3_-induced rate of irregularities in the outer retina (Fig. 5d).

## Discussion

MNU and NaIO_3_ have retinal specific toxicity, and we selected H_2_O_2_ in consideration of not only AMD but also its potential application to other diseases involving oxidative stress damage. H_2_O_2_ is a typical oxidative stress inducer and is used for *in vitro* evaluation of many useful substances. NaIO_3_ is a stable oxidant that selectively damages the retinal pigmented epithelium following its systemic delivery, resulting in retinal degeneration. The retinal toxicity of NaIO_3_ has been demonstrated in various animal species, including sheep^24^, rabbits, rats^25^, and mice^26^, using various doses and administration routes. NaIO_3_ has been used widely to evaluate neuroprotective treatments in preclinical animal models of retinal degeneration and geographic atrophy^27^. As shown by Tsuruma *et al*., MNU elevated reactive oxygen species (ROS) in rod and cone photoreceptor cells and induces photoreceptor cell death *in vitro*, and Edaravone scavenged ROS production in a concentration-dependent manner, and reduced MNU-induced retinal degeneration in mice^28^. Edaravone might inhibit MNU-induced photoreceptor cell degeneration by its ROS-scavenging activity. Additionally, it is reported that FA or EF has ROS-scavenging activity and reduces ROS generation in synaptosome^17,29^. The retinal protective effect of FA and EF is considered to be similar to the above mechanism by their ROS-scavenging activity.

Pre-treatment with FA attenuated NaIO_3_-induced retinal damage more effectively than post-treatment with FA (Fig. 3c). The beneficial effects of phytochemicals, such as green tea catechins and chrysophanol, have been shown in retinal degenerative models induced with NaIO_3_ or MNU; however, they were administered orally before NaIO_3_ or MNU injection^22,23^. Yu *et al*. reported that a daily grape diet could prevent age-related retinal pigment epithelium actin damage and blindness in mice lacking the αvβ5 integrin receptor^30^. The beneficial effect of a daily grape diet was shown during young adulthood (3–6 months of age) or midlife (6–9 months of age), but not during later life (9–12 months of age). Daily prophylactic treatment with phytochemicals may be required so they could exert an inhibitory effect on the progression of retinal degeneration. As for the inhibitory effect against NaIO_3_-induced retinal degeneration, EF showed a slightly weaker effect compared to FA, though EF has greater cell membrane permeability than FA. It is considered that some causes of this effect might include differences in their bioavailability. Kikuzaki *et al*. reported that the radical scavenging activity on 1,1-diphenyl-2-picrylhydrazyl (DPPH) decreased in the order ferulic acid > ferulic acid esters, and the higher effectiveness of ferulic acid than its esters might be influenced by the difference in their partition coefficients in the n-octanol/PBS system explained by their affinity with lipid. Ferulic acid suppressed formation of hydroperoxide more than alkyl ferulates^16^.

FA can be quickly absorbed (T max = 24 min) and eliminated (t1/2 = 42 min), and its oral bioavailability is ~20% in humans^31,32^. The oral administration of (-)-epigallocatechin gallate reportedly inhibits retinal degeneration in rats^18^. As the blood−retina barrier permeability of FA and (-)-epigallocatechin gallate is relatively high, we assume that FA could reach the retina^33^. FA and EF have free radical scavenging activity and can increase superoxide dismutase and catalase activity to promote the detoxification of reactive oxygen species. They can upregulate the expression of heat shock protein 70 and heme oxygenase-1 to exert a cytoprotective effect on damaged cells^13^. FA inhibits the production of inflammatory cytokines, such as IL-6, IL-1β, and tumor necrosis factor-α, in various animal models of hepatocytic and aortic inflammation and ulcerative colitis^34^. The representative mechanism of anti-inflammation is attributed to the ability of hydroxycinnamic acids including FA to inhibit the NF-κB pathway and suppress the expression of COX-2 and iNOS^35^.

It is considered that the therapeutic potential of FA includes antioxidant, antihypertensive, antihyperlipidemic, vasodilation, and hypoglycemic effects in cardiovascular disease^34^. The oral administration of FA reduces the levels of triglycerides, cholesterols, and blood pressure in hypertensive rats, and the reduction of blood pressure is observed at 1–2 h after administration^36^. In diabetic rats, oral FA treatment restores blood glucose and serum insulin levels and insulin and glucose tolerance to their normal range^37^. Chowdhury *et al*. showed that FA is sufficiently potent to provide protection to the heart from oxidative and endoplasmic reticulum stress by circumventing hyperglycemia, ameliorating cardiac damage markers, regulating the intracellular redox balance, and attenuating the eIF-2a- and caspase 12-activated endoplasmic reticulum stress-induced apoptotic pathway in streptozotocin-induced diabetic rats^38^.

AD is a neurodegenerative disorder characterized by the progressive loss of memory, cognitive dysfunction, and mood disorders. The pathogenesis of AD is associated with the formation of senile plaques consisting of aggregates of amyloid β (Aβ). FA inhibits Aβ monomer-to-oligomer transition and the formation of Aβ fibrils^39,40^. In many AD rodent models, FA attenuates neuroinflammation and improves some dementia-like symptoms related to cognitive performance^13,41,42^. Because the intravenous administration of FA has protective effects against the neuronal cell death induced by cerebral ischemia in rats^43,44^, it is speculated to be able to cross the blood-brain barrier^34^.

AD shares similar environmental risk factors with AMD, including smoking, hypertension, hypercholesterolemia, and an unhealthy diet^14^. Both diseases share similar cellular pathology, including oxidative stress and inflammation, and the detrimental deposition of Aβ in the ocular drusen and senile plaques in histopathologic features^45^. In contrast, some epidemiological research data provide evidence that there is no positive association between AMD and dementia or AD^46^. The oral administration of FA for 6 months was shown to reduce Aβ deposition in an amyloid precursor protein and presenilin 1 double transgenic mouse model of AD^41^.

To ingest FA, a diet of whole grains and vegetables and supplements of FA are suitable approaches. FA is the main metabolite of γ-oryzanol, which has antiinflammation and antioxidation effects, and was marketed under the name “Hi-Z” by Otsuka Pharmaceutical Co., Ltd. in 1970 for the treatment of hyperlipidemia and psychosomatic disorders (climacteric disturbance and irritable bowel syndrome)^47^. There are sufficient data available for previously marketed drugs such as Hi-Z on their pharmacokinetics, metabolism, and toxicity in humans. Drug repositioning is the process of discovering new uses for marketed drugs in a rapid and cost-effective manner and has become a popular strategy for drug discovery and development in recent years^48^. Daily dietary improvements, intake of FA supplements, and prescription of Hi-Z for repositioned indications might be able to slow age-related functional decline in AMD and AD.

Japanese people traditionally ate poorly-refined rice with various millets as a staple food containing various and rich antioxidants such as FA and γ-oryzanol until the Western diet was adopted widely in Japan. The traditional Japanese diet is associated with a lower prevalence of cardiovascular disease and AMD and increased longevity^49,50^. The traditional Japanese diet and the Mediterranean diet are representative healthy diets that have many common characteristic components such as whole grains, large portions of vegetables, fruits, legumes, and fish, and small portions of meat. Higher adherence to the Mediterranean diet is associated with a lower risk of incident-advanced AMD, and the traditional Asian diet is significantly associated with a reduced risk of early and advanced AMD. In contrast, the Western diet, which is characterized by a high intake of red meat and high-fat dairy products, is significantly associated with an increased risk of early and advanced AMD^5,51^. The recommended supplements for AMD include a combination of antioxidants (vitamins C and E, lutein, and zeaxanthin). However, in individuals who are reluctant to improve their diet, get more exercise, or quit smoking, the daily use of these recommended supplements might not be associated with a decreased risk of AMD. A healthy daily diet containing various whole grains and vegetables rich in antioxidants might be protective against AMD, slow the progression of AMD and other age-related diseases, and be associated with increased longevity^50^.

The mechanism by which an improvement in diet and lifestyle reduces the risk of AMD is unclear. Rowan *et al*. demonstrated that the consumption of a high-glycemia (HG) diet resulted in many AMD features in aged mice compared with a low-glycemia (LG) diet and that switching from the HG to the LG diet arrested or reversed the AMD features^52^. The composition of the gut microbiota is different between HG and LG diets, and the LG diet was shown to induce the production of metabolites that protected against AMD, including serotonin, by the gut microbiota. The traditional Asian diet and the Mediterranean diet contain fermented foods such as miso and cheese. FA is linked with a variety of carbohydrates such as glycosidic conjugates in grains. The carbohydrates covalently conjugated to FA can be administered orally, after which they are enzymatically hydrolyzed by the gut microbiota or liver metabolism and released in their free form. Lower-glycemic diets containing whole grains such as brown rice as a staple food can confer the daily recommended intake of FA and would help to protect against the development of AMD.

## Methods

### Materials

7KCh and MNU were purchased from Sigma–Aldrich Japan (Tokyo, Japan), MNU (containing water) from Toronto Research Chemicals, hydroxypropyl-β-cyclodextrin (HPBCD) from Cayman Chemical (Ann Arbor, MI), LPS from Wako Pure Chemical Industries (Osaka, Japan), NaIO_3_ from Nacalai Tesque (Kyoto, Japan), FA and EF from Tokyo Chemical Industry (Tokyo, Japan), and other reagents from Nacalai Tesque or Sigma. All reagents were “reagent grade.” After conducting the second animal experiment (Supplementary Fig. S2), MNU was discontinued at Sigma-Aldrich and no longer available.

### Cell culture

Human retinal pigment epithelial cell line ARPE-19 cells (American Type Culture Collection, Manassas, VA) were cultured in a humidified atmosphere (37 °C, 5% CO_2_) in Dulbecco’s modified Eagle’s medium and F-12 nutrient mixture (Nacalai Tesque) supplemented with 10% heat-inactivated fetal bovine serum and 100 μg/mL penicillin/streptomycin. The cells were passaged by trypsinization every 2 to 3 days. Each experiment was repeated at least 3 times.

### Cell viability analysis

ARPE-19 cells plated in a 96-well plate were treated (1) with or without FA for 3 days; (2) with or without FA for 1 h before a 1-h exposure to 0.3–0.4 mM H_2_O_2_ with fresh medium replaced every day for 3 days; (3) without drugs for 1 h before a 1-h exposure to 0.3–0.4 mM H_2_O_2_ with fresh medium containing FA or none replaced every day for 3 days. At the end of the incubation period, cell viability was assessed by WST-8 solution (Cell Count Reagent SF; Nacalai Tesque). The cells were incubated with WST-8 solution for 1–2 h and scanned at 450 nm with a microplate reader (Glow Max; Promega, Tokyo, Japan).

### ELISA

The level of secreted IL-6 in conditioned medium from ARPE-19 cell culture was measured at 48 h after treatment with 40 μM 7KCh complexed with HPBCD^53^ and 2.5 μg/mL LPS using an ELISA Duoset (R&D Systems, Minneapolis, MN). Protein levels were measured in triplicate.

### Experimental animals

All experiments were performed according to the ethical guidelines of the Kyoto University Animal Research Committee. The protocol was approved by the Kyoto University Animal Research Committee (permission number: Med Kyo 17082). All mice were maintained under a 14-h light/10-h dark cycle and fed *ad libitum*. Adult female C57BL/6 mice (weight, 25–30 g) were obtained from Nihon SLC (Shizuoka, Japan). Before ERG and SD-OCT image acquisition, the mice were anesthetized by an intraperitoneal injection of medetomidine, butorphanol, and midazolam (0.75, 5, and 4 mg/kg body weight, respectively). Pupils were dilated with tropicamide and phenylephrine eye drops (0.5% each).

### Pharmacological induction of retinal degeneration

Retinal degeneration was induced as previously reported^15,16^. Briefly, MNU or NaIO_3_ was dissolved in saline and injected interperitoneally at a dose of 60 or 40 mg/kg body weight, respectively. In the control group, the mice were injected interperitoneally with saline.

### Administration of FA and EF

We measured the amount of water that the mice drank with *ad libitum* access in pilot experiments and prepared FA and EF solutions (water containing 0.2 mg/mL FA or EF) aiming for the administration of 30 mg/kg/day of FA or EF. The mice were given oral FA, EF, or water only using a water bottle from 3 days before NaIO_3_ injection to 2 weeks after it.

### ERG

ERG using a gold-loop corneal electrode with a light-emitting diode (Mayo Corp., Inazawa, Japan) was performed^54^. A reference electrode was placed in the mouth, and a ground electrode was placed in the tail. Stimuli were produced with a light-emitting diode stimulator (Mayo Corp.). Then, the ERG response signals were amplified (PowerLab 2/25; AD Instruments, New South Wales, Australia). The photopic ERG responses of b-waves elicited by light at an intensity of 30 cds/m^2^ were recorded at 2 weeks after NaIO_3_ administration. B-wave amplitudes were analyzed using Chart & Scope software (AD Instruments).

### SD-OCT acquisition and measurement of retinal thickness

SD-OCT examinations using Multiline OCT (Heidelberg Engineering, Heidelberg, Germany) in C57BL/6 mice were performed at 2 weeks after NaIO_3_ injection. A 25-diopter adaptor lens was placed on the objective lens of the Multiline OCT to focus on the mouse retina. Outer retina thickness was measured from the outer plexiform layer until the retinal pigmented epithelium. However, as choroidal structures and photoreceptor layer damaged by NaIO_3_ cannot be reliably detected in pigmented retinas such as those of C57BL/6 mice^55,56^, we evaluated structural degeneration as the thickness of the ORC instead of the outer retina. The ORC was measured from the outer plexiform layer until the choroid using linear scan images near the optic nerve head. The resulting SD-OCT images were imported into ImageJ software (National Institutes of Health, Bethesda, MD) to measure ORC thickness.

### Histological analysis

On post-injection day 15, the mice were sacrificed by cervical dislocation. Their eyes were removed and fixed in SUPER FIX rapid fixative solution (Kurabo, Osaka, Japan). Fixed eyes were paraffin-embedded 5-μm sections were stained with hematoxylin and eosin (HE). Tissue section preparation and HE staining were entrusted to the Kyoto Pathology Laboratory. The images were captured using a BX-51 microscope equipped with a DP-25 CCD camera (Olympus, Tokyo, Japan).

### Statistical analysis

Statistical differences were determined using the unpaired Student’s *t*-test between two groups, or analysis of variance and then Dunnett’s test for the comparison of multiple groups by JMP Pro Ver.12 (SAS Institute, Cary, NC). *P*  <  0.05 was considered significant.

## Supplementary information

Supplementary information.
