# Supplementary material for: Oral administration of ferulic acid or ethyl ferulate attenuates retinal damage in sodium iodate-induced retinal degeneration mice
Source: Sci Rep. 2020 May 26;10:8688. doi: 10.1038/s41598-020-65673-y (PMC7250827; doi:10.1038/s41598-020-65673-y)
Supplement: Supplementary file 1 — Supplementary information. [file 41598_2020_65673_MOESM1_ESM.pdf]

Supplementary information

Oral administration of ferulic acid or ethyl ferulate attenuates retinal damage in sodium iodate-induced retinal degeneration mice

Masayuki Kohno, Kunihiro Musashi, Hanako Ohashi Ikeda, Tomohisa Horibe, Aki Matsumoto, & Koji Kawakami

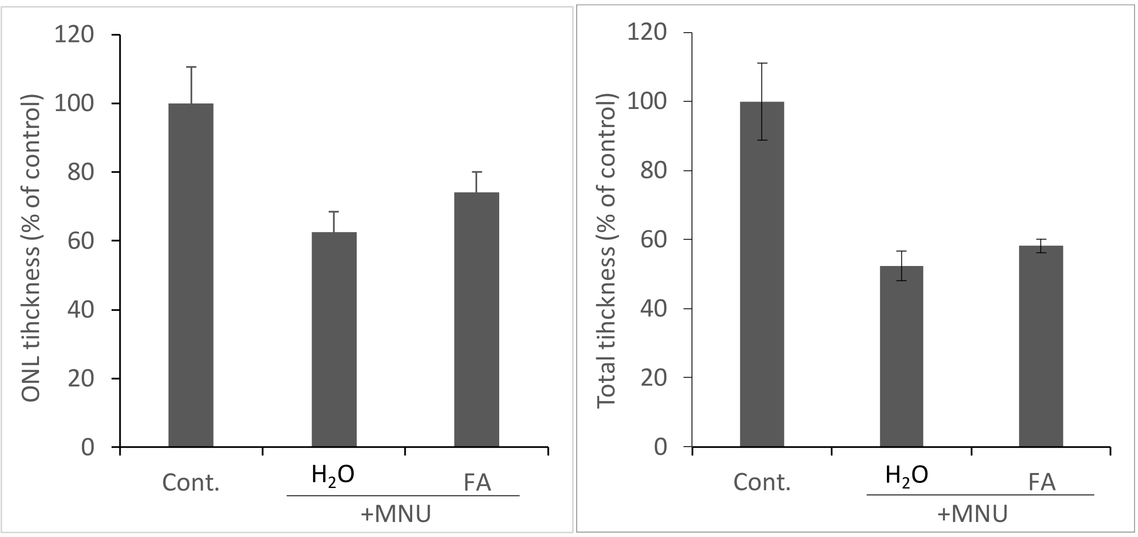

Supplemental Fig. S1. Oral FA attenuates MNU-induced retinal degeneration in mice (first experiment).

Vertical thickness of the outer nuclear layer (ONL, left) and total retina (right) in mice at 4 days after MNU injection (70 mg/kg) was quantified on histological sections. Data are shown as the mean ± SD (3 eyes/group). Cont., control.

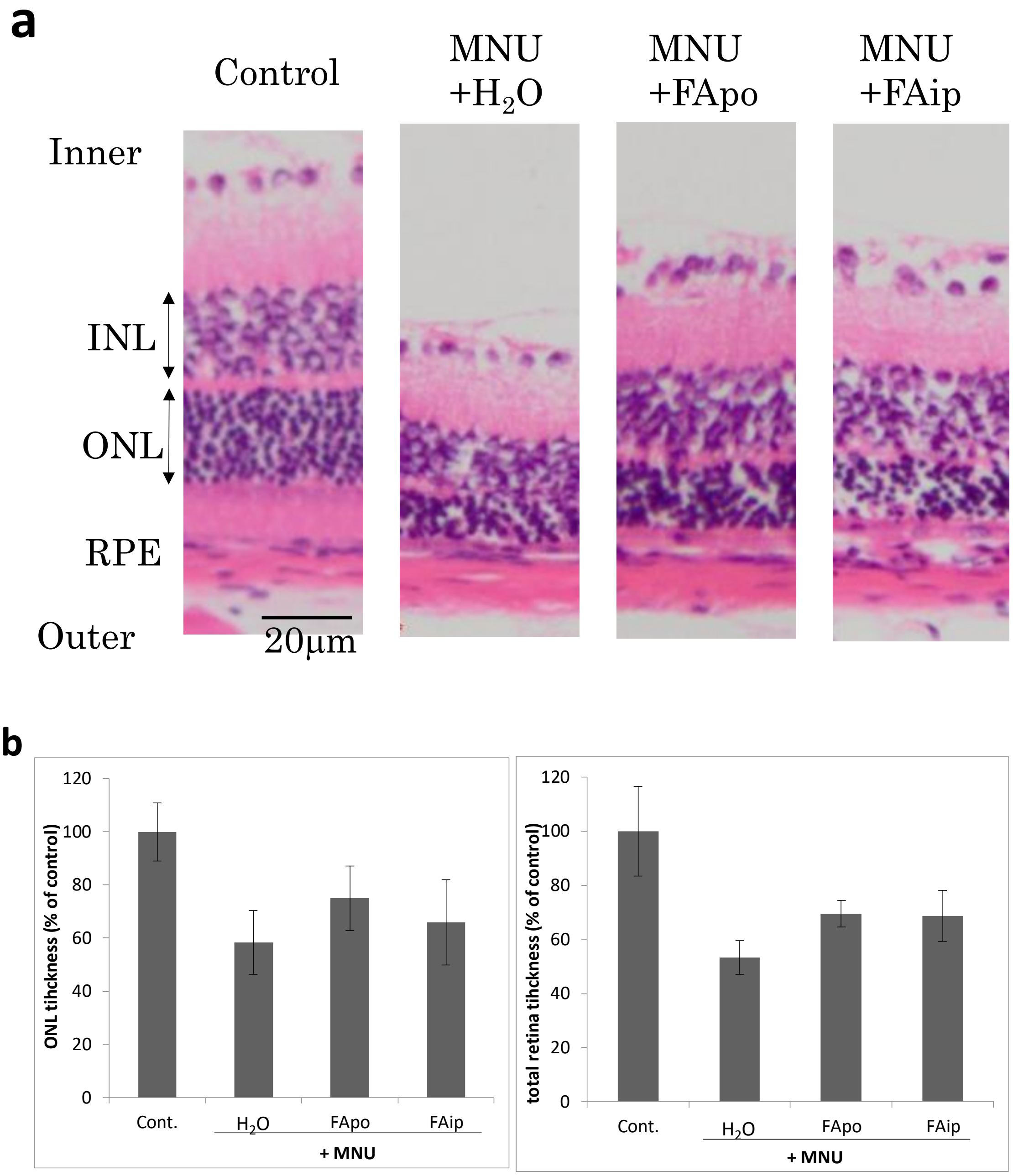
a
Control
MNU +H2O
MNU +FApo
MNU +FAip
Inner
INL
ONL
RPE
Outer
20µm
b
ONL tihckness (% of control)
120
100
80
60
40
20
0
Cont.
H2O
FApo
FAip
+ MNU
total retina tihckness (% of control)
120
100
80
60
40
20
0
Cont.
H2O
FApo
FAip
+ MNU

Supplemental Fig. S2. FA attenuates MNU-induced retinal degeneration in mice (second experiment).

(a) Representative microscopy images of normal retina (control mice) and retinas treated with various combinations of H_2O or FA for 4 days after MNU administration (60 mg/kg). INL: Inner nuclear layer, ONL: Outer nuclear layer, RPE: Retinal pigment epithelium.

(b) Vertical thickness of the ONL (left) and total retina (right) in mice at 4 days after MNU injection was quantified on histological sections. Data are shown as the mean ± SD (3 eyes/group). Cont., control; po, oral administration; ip, intraperitoneal administration.

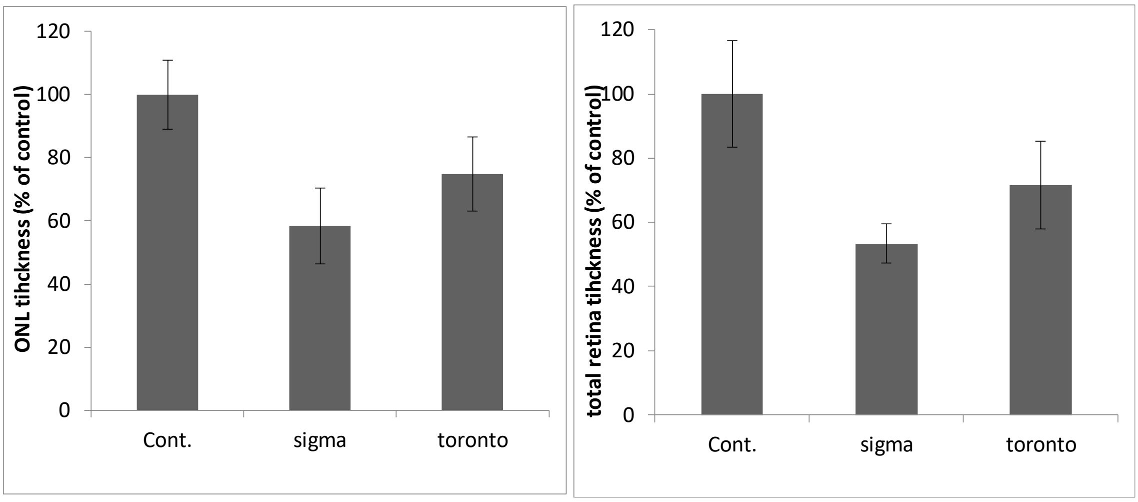

Supplemental Fig. S3. Comparison of the damage activity in retina by two MNUs. Vertical thickness of the ONL (left) and total retina (right) in mice at 4 days after MNU (Sigma-Aldrich or Toronto Research Chemicals) injection was quantified on histological sections. Data are shown as the mean ± SD (3 eyes/group). Cont., control.

Supplementary Table S1

The experimental design for effect of pretreatment or post-treatment of FA in $NaIO_3$-induced mice model.

Treatment	pre-oral	ip injection	post-oral	
Days	-3～1	0	1～15	eyes
Control	H_2O	saline	H_2O	4
H/H	H_2O	$NaIO_3$	H_2O	8
F/F	FA	$NaIO_3$	FA	8
F/H	FA	$NaIO_3$	H_2O	8
H/F	H_2O	$NaIO_3$	FA	8

FA (40mg/kg) or H_2O was orally administrated using water bottle for total 18 days. ip:intraperitoneal.

Supplemental methods

Pharmacological induction of retinal degeneration

MNU was dissolved in saline and injected interperitoneally at a dose of 70 or 60 mg/kg body weight. In the control group, the mice were injected interperitoneally with saline.

Administration of FA

FA (10 mg/kg/administration) was administered orally using a feeding needle once or twice a day, or interperitoneally once a day from the first day of MNU injection to 4 days after it.

Histological analysis

The mice were sacrificed by cervical dislocation at 4 days after MNU injection. Their eyes were removed and fixed in SUPER FIX rapid fixative solution (Kurabo, Osaka, Japan) and paraffin-embedded 5-μm sections were stained with HE. The images were captured using a BX-51 microscope equipped with a DP-25 CCD camera (Olympus, Tokyo, Japan).
